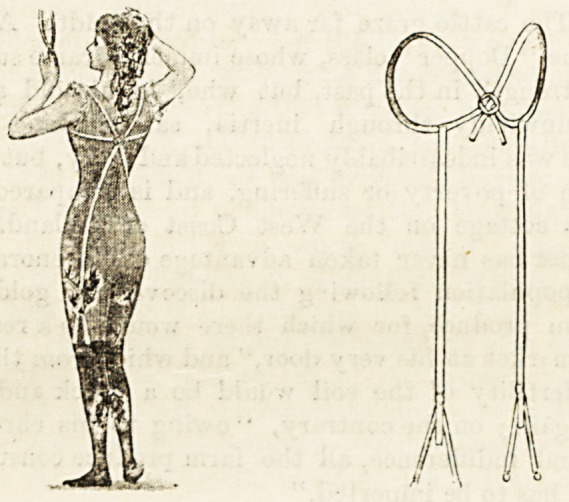# "The Hospital" Nursing Mirror

**Published:** 1900-02-17

**Authors:** 


### "The Hospital" Nursing Mirror.

Being the Nursing Section of "The Hospital."

[Contributions for this Section of "The Hospital" should be addressed to the Editor, The Hospital, 28 & 29, Southampton Street, Strand, London, W.C., and should have the word "Nursing" plainly written in left-hand top corner of the envelope.]

### Notes on News from the Nursing World.

### HOSPITAL SHIP THE "PRINCESS OF WALES."

A nurse on board the "Princess of Wales," writing to us from Cape Town under date of January 18th, says: We have not had any patients yet, and have been rather long coming out. I went up to Wynberg yesterday, and found it most interesting. Nos. 1 and 2 general hospitals are quite near, side by side. There are 600 beds at No. 1, and they are wooden houses, each ward separate, and they have one full of Boers. The sister told me that they make very good patients. No. 2 is all under canvas; each tent holds six beds, and there are 500 altogether; they are on the old cricket ground of the barracks. Some patients were expected when I was there. I went up by the electric tram nine miles, through pretty country the latter half. The flowers are splendid—plumbago hedges to most of the gardens, and such roses! The hospital is well supplied with comforts. One lady brought a custard, another rice pudding and eggs, some children flowers and strawberries. Fancy poor Tommy feeding on such goodies. We are now in Government Dock. It is very interesting watching the war appliances unpacked. Last Sunday, the 14th, we saw about 1,700 troops off. The dock station is quite near here; the troops came in in the "Gaika," and we saw them all start, including the Army Nursing Service Corps. We knew two of the officers, and sister packed a basket for them and put in the train, and a lot of papers in every carriage for the men. We were the only persons to see them off at half-past ten, and the men cheered us and hoped they would meet us again. No doubt they will some of them get injured, but they did not seem to think much of that. They were a fine-looking lot of fellows.

### THE WAR NURSES AND TYPHOID.

To be inoculated against typhoid and to be vaccinated seems to be essential on the part of all the nurses proceeding to South Africa. "So accordingly on Saturday evening," says one of the nurses of the Imperial Yeomanry Hospital, "I went to Cavendish Place, where lately Dr. Washbourn has been so busy doing all that modern science can suggest for the prevention of those dread diseases, typhoid and small-pox. First I was vaccinated, then came the more interesting operation of inoculation. The anti-typhoid vaccine was injected into the abdominal wall. It was soon over, and after a few directions from the doctor I left. Within two hours I was in bed with two hot-water bottles and piles of blankets —in spite of which I felt shivery—and for a couple of hours I alternated between a gooseflesh sensation and bursts of heat. My head ached violently for an hour, but was relieved by an attack of vomiting. Three hours after the injection my temperature was 100·4. Judging by my sensations later it rose an inch higher, but by that time my abdomen and the glands of the left axilla were so painful that I could not have made the necessary effort to reach my thermometer had I felt so inclined. The night seemed interminable, and no sleep came to shorten the hours (if a *patient's* word is worth anything on that point). The next day I remained in bed feeling pretty miserable. At night my temperature was again 100·4. I dozed fitfully for some hours, imagining myself at some Carnival, surrounded by grotesque figures, though really quite conscious that I was in bed, but fell into a sound sleep towards morning and wakened feeling much better. The second day I got up, and if it had not been for the abdominal tenderness I should have felt almost all right. However, this condition gradually improved, and left me contemplating the development of my vaccinated arm.'"

### A MELBOURNE NURSE WITH THE LADYSMITH GARRISON.

Among the nurses in the Military Hospital at Ladysmith is Miss Rose Shappere, who was trained at the Alfred Hospital, Melbourne. On arriving at Johannesburg she volunteered to go forward to the border with the Boer commando. She then went to Standerton, and subsequently got to Ladysmith just as the bombardment of the town by the Boers was going on. She has therefore enjoyed the experience of nursing on both sides. Her friends report that when they last heard from Miss Shappere she was "quite well, strong, happy, and cheerful," but has "lots of hard work."

### A FAREWELL FUNCTION AT THE NURSES' HOSTEL.

On Saturday last at the Nurse's Hostel a farewell tea was given by the three nurses belonging to the institution who have been selected for service in the Imperial Yeomanry Hospital. The opportunity was taken of presenting them with a number of useful articles which, as explained by Miss Paul, though selected by herself, had been subscribed to by many who made the Hostel their headquarters, and must be taken to represent the sympathy and interest of the nurses left behind with those who have had the privilege of being chosen to nurse the sick and wounded in South Africa. The selection proved the kindly thought and care which had been expended over it. There was a down cushion with two linen covers, a compact and beautiful nickel-plated Etna with an accompanying flask of methylated spirits, a hot-water bottle, one dozen handkerchiefs, box of soap, note paper and envelopes, and many other things which will often serve to remind the nurses of the kind friends at home. Miss Lancaster, on behalf of herself, Miss Walkinshaw, and Miss Gertrude Fletcher, in a few suitable words expressed their gratitude and appreciation of the gifts. The afternoon ended by Miss Wood first, amid much laughter, requesting the three nurses to stay behind and allow the others present to go in their stead. She then called upon them to prove once again what English women could do at such a time, and in a few touching words commended them to the care of a Higher Power.

### THE METROPOLITAN ASYLUMS BOARD AND MISS ATKINS.

It is quite true, as Mr. G. L. Bruce said on Saturday at the meeting of the Managers of the Metropolitan Asylums Board, that it is no more heroic to nurse the sick and wounded in South Africa than to take charge of one of the Board's hospitals. But at a time when every man or woman who is considered to be specially qualified to be of service either in defending the interests of the Empire, or in ministering to its defenders, is naturally proud to be selected for duty, it seems ungracious for any public body to place obstacles in the way. For this reason we regret that the majority of the Asylums Board, throwing over the decision of the previous fortnight, have finally declined to grant Miss Atkins the leave of absence which she desired to obtain, without pay, in order to enable her to proceed to one of the military hospitals at Cape Town. She is going in spite of this determination, but the refusal means, of course, that her post at the Park Hospital will be permanently filled up.

### THE ILLINOIS TRAINING SCHOOL FOR NURSES.

We are able this week to afford our readers the opportunity of perusing an article by Miss Isabel McIsaac, the superintendent of the Illinois Training School for Nurses at Chicago, one of the greatest training schools in the United States. A remarkable feature in connection with the Illinois school is that the nurses tend the sick of two large hospitals, "each worked upon an entirely different basis from the other." Yet there has been no serious friction for twelve years. Miss McIsaac deals with the advantages of the arrangements in respect to the training, one of which is that the Presbyterian Hospital gives the best of training in the case of private patients. The Cook County Hospital has an extraordinary number of acute medical cases. It will be observed that the conditions of admission vary considerably from those of training schools in this country, and that the schedule of time for the three years is different to ours.

### A PIONEER OF THE NURSING MOVEMENT.

The late Mrs. Stuart Wortley, who was buried on Friday, was a staunch friend of the nursing interest. She was not only one of the originators of the East London Nursing Society, but she was also a promoter of the Metropolitan Nursing Association. It was largely in consequence of her efforts that the late Duke of Westminster was persuaded to take up the movement and carry it to a successful issue. Mrs. Stuart Wortley played the part of a benefactor of the poor during the cholera epidemic in London in 1868, and it was in the course of these experiences that she and two of her friends organised the first society of the kind for nursing the poor in their own homes. She was a strong advocate of training, and rejoiced greatly to see the fulfilment of some of the aspirations which she entertained in early days.

### THE MATRON OF ST. GEORGE'S INFIRMARY, FULHAM ROAD.

We print with pleasure a letter from two nurses of St. George's Infirmary, Fulham Road, who desire to commend the manner in which the matron discharges her duties. But with regard to the suggestion that we have spoken "slightingly" of that lady, we must point out that we have most carefully refrained from saying anything disparaging about her. We should not have mentioned her at all if it had not been for the report that she had resigned. The two nurses describe that report as "somewhat premature." We can only express the hope that if the matron remains at St. George's Infirmary she will show that she is as able, as it appears she is willing, to ensure proper treatment for the patients, and we gladly give her credit for the concessions which have been made to the nurses since our correspondent took up the cudgels on their behalf.

### MORE INADEQUATE INFIRMARY NURSING STAFFS.

St. George's Infirmary is not the only workhouse infirmary in London whose inmates have suffered from the practice of a false economy. At an inquest which was held at the Holborn Union Workhouse the other day on the body of an old woman who got out of bed while the nurse was in another ward, and burnt herself to death with the hot-water pipes, it was stated that two night nurses had to look after 228 patients. This is a monstrous condition of affairs, and calls for an immediate alteration. It cannot be pretended that the Holborn Board of Guardians are short of funds; and yet the nursing staff is expected to perform utterly impossible duties. Their inevitable failure must now and again result in catastrophes, while it is quite clear that the patients in the Holborn Infirmary cannot at any time have proper attention. Every day there is fresh evidence of the necessity for a searching reform in the arrangements of workhouse infirmaries; and as we write there reaches us a letter, which we publish elsewhere, in reference to a North London infirmary that points to the existence of real grievances. But there is no hope of rectifying them so long as the name of the institution is not stated.

### SETTING ASIDE THE AGE LIMIT.

The Governors of the Northampton General Infirmary have been wise enough to set aside the age limit in the case of the greatly esteemed matron of that institution. Miss Pell, who has held the position for between 34 and 35 years, had reached the limit, and she reported the fact to the Governors. They, however, taking advantage of a special clause in the rules, asked her to remain. The compliment is, of course, a great one, and, apart from the personal aspect of the matter, the action of the Governors merits the highest commendation. As Miss Pell has filled an arduous office for so many years, with signal success, and is still as able to discharge her duties as ever, why should she retire, and the experiment of a new appointment be prematurely made? We hope that the example of the Governors of the Northampton General Infirmary will be followed elsewhere. If other rules have no saving clauses, they are not as unalterable as the law of the Medes and Persians.

### THE NEW NURSES' HOME AT GATESHEAD.

The handsome and comfortable new nurses' home at Gateshead, presented to the Nursing Association by Mr. and Mrs. Walter de Lancey Willson, was opened last week. The building, which is an adaptation of the domestic Gothic style, is constructed of brick, with facings of Denwick freestone, and the roof is covered with red Staffordshire tiles. On the ground floor are the sitting and dining rooms, on the first floor five good bedrooms, and on the second three large attics and a boxroom. Mr. Willson has always been a good friend of the nursing movement. He started the Nursing Association when he was Mayor of Gateshead, and having seen it become an important organisation, with a staff of five nurses, he has, in conjunction with his wife, provided admirable accommodation for those engaged in the work. As the town is growing at the rate of 2,500 a year, more nurses will soon become essential.

### ST. PATRICK'S NURSES' HOME, DUBLIN.

At the annual meeting of the St. Patrick's Nurses' Home, Dublin, the twenty-third yearly report was adopted. The staff now consists of lady superintendent and eight fully-trained nurses, with a constantly varying number of probationers receiving district training. Last year ten of these passed through the home, and have since been sent by the Queen Victoria's Jubilee Institute to different districts from which applications for nursing help were made. The number of cases nursed by the staff was 1,838, and the number of visits paid was 33,001. An encouraging fact noticed by the nurses in their daily rounds in Dublin is the improvement they see in the rooms they visit. Many letters expressing the gratitude felt by the poor for the assistance given in "tidying" things and in other little ways, come to the home, showing how deep is the appreciation of the benefits the writers and their friends have received.

### THE LONG HOURS OF MENTAL NURSES.

We are asked to put in a plea for the reduction of the hours of mental nurses, most of whom, it is stated by a correspondent, are on duty from six a.m. to eight p.m.; while the hours of the head attendants are from six to ten. In a workhouse for imbeciles in the West of England, with 114 beds, there are seven attendants, including the head, and two of these are on night duty. Formerly the inmates were allowed to assist, but since this has been forbidden by the Local Government Board the work of the attendants must obviously have become heavier. Bearing in mind the peculiar strain of nursing or attending the insane, a reduction in the hours seems to be urgently required. One effect of such a reform would probably be an improvement in the class of mental nurses, who, in existing circumstances, have too often not been properly trained.

### LEEDS TRAINED NURSES' INSTITUTION.

From the twenty-fourth annual report of the Leeds Trained Nurses' Institution, we learn that there are 99 nurses engaged in private nursing and 12 probationers in training in hospitals. In 1899 12 nurses left the institution, "some to be married, others for work elsewhere." After careful inquiry as to the custom elsewhere under similar circumstances, the committee considered it right last year to raise the salaries paid to the nurses substantially, a step which involved a proportionate rise in the fees for nursing. That this rise is not resented may be gathered from the fact that, though 1,047 cases were undertaken, no less than 350 had to be refused owing to no nurse being at liberty when application was made. The district nursing branch continues to increase in extent and usefulness, and there are now 18 districts covered by the work, 2,903 cases having been taken last year. The balance of profits has been appropriated between the Leeds Trained Nurses' Institution Trust Fund of the Royal National Pension Fund for Nurses and the Leeds District Nursing Association.

### THE "BROWN RITERS."

Among the poor the nomenclature of disease is decidedly unique. "The other day," writes a nurse in Suffolk, "I came across a rather amusing instance. I was talking to a woman in the district, and happened to inquire about the health of her daughter, who lives some distance away, and to whom she had been paying a visit." "Well, Miss," replied the good dame, "she fare (feels) very bad, and the doctor do say how she has got a rare lot o' complaints, 'enema' and a 'ulster' on the stomach, and the names of the others I forget; and her husband, he do suffer cruel with the brown riters this winter, he's had them on him since long afore Christmas."

### SHORT ITEMS.

Nurse McCaul, whose work in nursing the wounded at Chieveley was mentioned in the account of Mr. Treves, formerly lived at the old Nursing Institution in Nuneaton, and is the sister-in-law of a local solicitor.—Princess Christian has consented to become President of the Haggerston and Hoxton District Nursing Association (including parts of Bethnal Green, Hackney, and outlying districts), over which the late Marchioness of Salisbury had presided since its formation ten years ago. The association works in one of the poorest and most crowded parts of London, and the demand for the trained nursing it supplies is so large, and so continually increasing, that the committee are now making an urgent appeal for funds to enlarge the home, and fit it to accommodate 14 nurses.—Mrs. Scharlieb, M.D., will lecture, under the auspices of the Royal British Nurses' Association, at 11, Chandos Street, Cavendish Square, on Thursday next, at six p.m. Her subject will be "Nursing in Cases of Cancer of the Womb."—The nurses of the Scottish Hospital for South Africa, which is to be dispatched to the seat of war, will all be natives of Scotland.—Dr. Francis Warner will lecture, under the auspices of the Childhood Society, on "The Elements of Early Training," at the library of Sanitary Institute, 72, Margaret Street, on February 17th.—At the annual meeting of the Barrow District Nursing Association the other day the gratifying announcement was made that the operations of 1899 had resulted in a balance of £250 of receipts over expenditure.—The sixth of the series of entertainments provided during the winter months for the amusement of the in-patients at the Cancer Hospital was given on Thursday evening last by Mrs. Arthur Read and friends. Previous to the rise of the curtain Mrs. Read's little daughters distributed daintily-arranged button-holes to all the patients and nurses. The various items on the programme were much appreciated and heartily encored.—In the first issue of *Women's Employment*, by the Central Bureau for the Employment of Women, which contains a variety of useful information, there is an excellent article on "Upholstery as a Trade for Women in London.—On Friday evening, February 23rd, a lecture, entitled "The Medical Aspects of Modern Warfare," will be given by Mr. F. R. Humphreys, L.R.C.P., at the Trained Nurses' Club, 12, Buckingham Street, Strand. The admission to non-members will be 1s., the proceeds to be given to A.M.B. War Fund.

### Lectures on Medicine to Nurses.

By H. A. LATIMER, M.D. (Dunelm), M.R.C.S. Eng., L.S.A.Lond.; Consulting Surgeon, Swansea Hospital; Past President of the South Wales and Monmouthshire Branch of Brit. Med. Assoc.; Past President of the Swansea Medical Society; Hon. Life Member of the St. John Ambulance Association, &c.

### LECTURE I.

### MEDICAL DISEASES CONTRASTED WITH SURGICAL ONES—HEALTH—HEREDITY.

IN this and the ensuing lectures which I am about to contribute to THE HOSPITAL I propose to follow the lines I adopted in a previous set which I addressed to surgical nurses. I purposely entitled the previous series "Lectures to Surgical Nurses," and refrained from describing them as lectures on surgical nursing: for although I dealt with the especial duties which would fall to a nurse in attending on a surgical patient, my object in delivering them was not so much the giving of instruction in the art of nursing as the teaching in a plain manner some of the facts about disorders of a surgical nature which I felt would be useful and interesting to those of you who might be attending patients suffering from diseases of that kind.

I feel that the true art of nursing can best be taught by practical work in the wards of a hospital or in the sick room, and that its best teachers must necessarily be the superintendents and sisters who, after having passed through a course of training such as you are undergoing, have performed the practical duties of ward nurses before they have attained the higher ranks. From them you become instructed in the technique of your calling in the only way in which such instruction can be thorough and efficient.

But, apart from such training, it is desirable that you should acquire some knowledge of the diseases to which we are all liable; and the object of the present lectures is to impart this on the subjects which are embraced by the title of medical diseases. The fixed line of demarcation which formerly separated the physician from the surgeon is now being rapidly swept away by the advances of science and by the practical fact that in modern plans of treatment the latter is constantly employed where formerly the physician only was consulted. As surgery becomes bolder in its methods the tendency is more and more shown for its application in the cure of diseases; and so it results that the internal cavities of the body which were formerly relegated to the care of the physician, and were sacred from the surgeon's knife, are now as often treated for disease by operative measures as by the administration of medicines. We may cite as an illustration of this fact the various disorders affecting the abdominal viscera. The time is not so long distant since persons suffering from gall or kidney stones came entirely under medical treatment for the cure of these distressing and dangerous troubles. Now, if relief to their sufferings is not soon effected by drugs the surgeon's aid is called in, and I might multiply examples of the same fact drawn from diseases affecting the contents of the skull, the thorax, and the abdomen were it necessary for me to do so. It suffices for me to notice it simply and then to pass on to the subject of these lectures.

What, then, are medical diseases? The answer, I take it, must be the arbitrary one that they are those ailments which are dealt with by the administration of drugs and by the regulation of air, diet, and exercise. The class must necessarily be a shifting one as methods of treatment vary from time to time, and there must be a borderland of diseases which may legitimately be claimed by the physician and the surgeon as common property. In these lectures I shall restrict the term to apply to those complaints which do not call for surgical interference in any other way than for the treatment of some complication which has set in and which has really nothing to do with the essential nature of the main disorder which is under consideration. Such a list will embrace the specific fevers, such as enteric and rheumatic fevers; constitutional disorders, such as gout and rheumatism; and affections of internal organs, such as the heart, lungs, kidneys, &c. The list which may be embraced under such headings is a very extensive one, and there can certainly be no lack of material for an author to deal with.

It will not be amiss, in the first place, to devote a little time to considering what is comprised by the term health, the condition of more or less perfection from which we ought to start the journey of life. I say "ought" advisedly, for it is unfortunately a fact that many people are handicapped in running the race of life by the fact that they do not, even from the beginning of it, have things arranged fairly for them in this respect. We all know what is comprised by it, and understand that it means a state of bodily condition which is free from disease; that a person in health has the various parts of his bodily machine performing their appropriate functions in a hale and sound manner. The various parts of the body are so intimately connected, and their functions are so interwoven, that the general health of the system depends upon the individual health of its several parts. If, for example, one organ, like the stomach, fails in its duty of dealing with the first part of the digestion of foods. Disease soon shows itself in one form or other, and the conscious self of its owner is disturbed and distressed. And so it happens that the first time, in many cases, that we know anything about our health is when the same is disturbed by some pain or disability which affects us. The priceless boon of a hale and sound state is often bartered away for some possession such as wealth and position in life which, when attained, is unenjoyable, because, in the efforts made to obtain it, the organism has been overtaxed and the capacity for pleasure has left us. And still more often is it lost by the indulgence in baneful habits which wreck the very tissues of organs within us, and in the end bring the action of the bodily machine to a standstill by death.

So it happens that the most beneficial part of a physician's work is often found to be that which lies in instructing his patients how to conserve the good health which they may have started with, and in telling them how to live so that they may maintain such a stock of it as they happen to possess when they consult him. It is true that this is not the popular conception of his duties. The general impression among the uneducated is that he is a person who is trained to discover the nature of an illness that is presented to him, and that, having made this discovery, he has only to apply to this entity (the disease) certain drugs which possess the certain action, if appropriately chosen, of bringing about a cure of it. It is an extraordinary fact how widespread this belief in the existence of "specifics"—by which is meant medicines having an absolute and selective action on certain diseases—is; we meet with it in all classes of life, even amongst people who by their superior general education would be supposed to have a better conception of what life is, and of what disease consists of. It is at the bottom of the great financial success achieved by the quack pill and medicine vendor, who, by skilfully availing himself of the prejudice, is enabled to reap a great fortune from the public. Such a person never has any doubt of the power of his vaunted specific over the disease he has introduced it to a credulous public as being the cure for. His patent pill for the cure of gout can never fail! Nor does it—to bring money into his pockets, provided he is content to sufficiently advertise it. And yet, on the face of the matter, it is an absurdity, for a moment's reflection must tell anyone that a disease like gout must have a hundred causes, and that the cure of it cannot lie in the administration of any one or two drugs. No! the rational and successful treatment of disease, where by the nature of it a cure is possible, is, with a very few exceptions, not effected by drugs having specific actions. In that part of the treatment where drugs are concerned these mostly act in an ascertained manner by aiding Nature in her work, in a manner which I hope to speak of at a later period of these lectures. When the appropriate time for dealing with any of the specifics presents itself I will deal with them, and will point out to you that even these have their limitations of usefulness.

### The Illinois Training School for Nurses, Chicago, U.S.A.

By Isabel McIsaac, Superintendent.

The establishment of the Illinois Training School for Nurses opened the history of trained nursing in the West, being the first of its kind in the great city which has made such phenomenal strides in commerce and educational institutions.

In the autumn of 1880 a group of philanthropic women met to confer upon the subject of establishing such a school, first to provide better nurses for the sick in their own homes, and, further, to open the way to young women desiring to fit themselves for self-support.

The origin of most of the training schools has been in the efforts of persons connected with hospitals to make better provision for hospital nursing, but in this instance the improvement of the hospital did not fully dawn upon the founders until the school was well started.

Not one among these women was a doctor or a nurse, but they knew that Cook County Hospital was a large public institution with an excellent medical board, spacious wards, and numerous patients, affording every variety of disease to be studied, and therefore in this hospital they resolved to plant their school.

Cook County Hospital provides for the sick poor of Cook County, in which Chicago is included, or, one might say, Chicago includes Cook County, the boundary of the former nearly, if not quite, covering the latter. The hospital is governed by a board of County Commissioners elected by ballot every two years.

To anyone at all conversant with our municipal politics it may readily be seen that the design of the training school board met with much opposition from the County Commissioners, who argued that the old-time nursing was good enough. For some time the ladies pleaded in vain, but an election carried off some of the objectors, and the new members were more reasonable.

A formal proposition was made to assume charge of two wards, medical and surgical, the county to pay the school the same sum paid to the women hitherto employed as nurses, and the school to provide its pupils with a home. It was further set forth that there was no doubt the experiment would be successful, and would entail no extra expense upon the Commissioners. This proposal was finally accepted by making a contract, which is renewed annually.

### The Start and Development.

An appeal was made to the public for funds, and met with generous response. A small house was taken in the neighbourhood, and on May 1st, 1881, the superintendent, Miss M. E. Brown, with two assistants, all from Bellevue Training School, New York, and eight probationers, assumed their duties in the two wards assigned to them, and the Illinois Training School for Nurses became a living fact. The school was modelled after Bellevue, which furnished it with superintendents for fourteen years. Among these were Miss Hampton, Miss Draper, and Miss Dock, nurses well known for their admirable work. The writer, a graduate of the school, has been in charge since 1895. Theoretical instruction by means of classes, lectures, and demonstrations were started at once, following the lines of the practical training.

The annual contract stipulates that the school shall provide a certain number of nurses for a certain sum, the discipline, teaching, and arrangement of nurses' time being entirely in the hands of the superintendent of the school. This very important fact has kept the school out of any political influence so detrimental to the proper training of nurses, and accounts largely for the steady, healthy growth which has never ceased. The annual report of October, 1882, states remarkable progress, the school having charge of the children's and all female wards, and numbering 22 nurses. The third report gives an increase of 33 nurses, and it became necessary to provide a permanent home. Again the public generously subscribed funds whereby land was purchased within half a block of the hospital and a commodious three-storey brick house was built.

The fourth year the nurses had increased to 45, and from that time until the present the growth has gone on steadily, until it now numbers 180.

In 1888 the school undertook the nursing in the Presbyterian Hospital, a private institution then having 60 beds—since grown to 225—whose medical board is made up by the faculty of Rush Medical College.

Prior to this time pupils had been sent out into private families for training in the care of private patients, and while this had been a source of revenue to the school, the disadvantage to the nurses was very great because of the interference in a systematic course of training. Since this connection with the Presbyterian Hospital the custom has been entirely abolished, and thereby pupils may finish both theoretical and practical work without interruption.

The same arrangement regarding teaching, discipline, and the nurses' time was made with this hospital as with the Cook County Hospital.

### How the Arrangement Works.

The question is very often asked, How does this arrangement work? Here is a school, entirely independent in government and finances, caring for the sick of two large hospitals, each worked upon an entirely different basis from the other. How can it be done satisfactorily to all concerned? My answer is, that it has been done for twelve years with no serious friction.

It is an economical arrangement for both institutions, the school being duty bound to give good service, amenable to the rules governing the hospitals. The advantage to the nurses is great; first, in providing a separate home, which has grown with the size of the school, until it now contain not only living and bed rooms, but also school rooms, a diet kitchen for class work, and its own little hospital for the care of sick pupils.

The board of managers give their interest and time to the school alone. The teaching, discipline, health, and home life are entirely under their direction, creating a much more educational atmosphere than in schools where the nurses are regarded more as a means of accomplishing the hospital work.

It is still further agreed by outsiders that this arrangement leaves the way open for great division of opinion and interests. My only answer to this argument is, that after many years of observation, I have frequently known of far more trouble and division in one year, between boards of managers and superintendents of training schools, in hospitals having their own schools, than our system will show for the twelve years.

Another great advantage is in the training afforded by the two entirely different hospitals, the Presbyterian giving the best of training in the care of private patients, and the Cook County having an extraordinary number of acute medical cases.

During the winter the wards are filled with large numbers of cases of pneumonia, rheumatism, uræmia, and other diseases peculiar to our damp winters, and in the summer with typhoid and malarial fevers, as well as many sun strokes and heat prostrations. Added to this is a contagious department where the training is optional; about five-sixths of the pupils taking it.

The faculty of the school consists of a superintendent, four assistants, two in each hospital; two night superintendents, one in each hospital; one secretary of the directory for graduates, and ten graduate head nurses in charge of operating rooms, contagious, children's, maternity, and private wards; the smaller and less important wards being in charge of pupil head nurses who are each given five months of this service during their third year.

The superintendent and four assistants conduct all classes and demonstrations which are carried on daily, the lectures being given by members of the medical staff of both hospitals.

THE CONDITIONS OF ADMISSION.

The following are the conditions upon which pupils are admitted:—

The Board of Directors of the Illinois Training School for Nurses, connected with the Cook County and the Presbyterian Hospitals, offers a three years' course of training to women who desire to enter the profession of nursing. The course of instruction comprises: Practical work in the wards; theoretical work in class and lecture; lessons in cooking; training school administration; and is divided into the junior, middle, and senior years, as follows:—

JUNIOR YEAR.—Class work: Elementary anatomy and physiology, Materia Medica, and practical nursing, embracing the whole care of ordinary medical, surgical, and gynæcological patients, with text-books, models, and demonstrations. Lectures: On hygiene, anatomy, and physiology; on Materia Medica and bacteriology; on surgical, medical, and gynæcological nursing. Cooking lessons: Practical and theoretical work.

MIDDLE YEAR.—Class work: Obstetrical nursing, care of the newborn, care of children, special nursing, care of operation patients, private duty, surgical technique, and operating-room work. Lectures: Obstetrics, special gynæcological work, the care of sick children, the examination and testing of urine, care of the nervous and insane, advanced medical and surgical subjects, eye, ear, massage.

SENIOR YEAR.—No class work. Lectures: Public Hygiene, Training-School Administration. During the senior year nurses will serve as head nurses and special nurses to private patients in the Presbyterian Hospital. Text-books in use: Kimber's "Anatomy and Physiology," Hampton's "Nursing," Dock's "Materia Medica for Nurses," Boland's "Cooking for the Sick." In addition to the text-books furnished, the school provides a large reference library. The practical work in the wards follows the same lines and is continuous throughout the three years' time. Classes and lectures begin the first week in October and last until the end of May, with the usual intermission at Christmas and Easter. Junior and middle year examinations are held in September, senior examinations in May, graduating exercises in the first week of June. Applications must be made to the superintendent of the training-school, 304, Honore Street, Chicago. The applicant is required to fill out the answers to the paper of questions, and to send with it a letter from a physician and one from a clergyman, testifying to her physical and moral qualities. If suitable, she is then received one month on probation. The most acceptable age is from 23 to 35 years. Applicants may be received at any time during the year when there is a vacancy, but it is greatly preferred that they enter not earlier than March 1st and not later than November 1st, as thus the entire course of study may be taken without interruption. During the probation month an examination in reading, penmanship, simple arithmetic and English dictation is given. The superintendent has full power to decide as to the fitness of the applicant for the work, and to retain or dismiss her at the end of the month of probation. In doubtful cases the time of probation may be extended to three months. Upon being accepted as a pupil nurse the candidate is required to sign an agreement, promising to remain for three years, and to conform strictly to the discipline of the school and hospital, with the distinct understanding that the Board reserves the right to dismiss her at any time for misconduct or inefficiency. If for any reason of her own, illness excepted, the pupil breaks this agreement and leaves the school she is required to refund to it the money expended for her maintenance. During the probation month, board, lodging, and laundry work are provided by the school. The probationer provides her own dress and may not wear the school uniform. After having been accepted as a pupil, uniform and text-books are furnished in addition. During the senior year pupils receive ten dollars per month. The hours of work are nine hours day duty and twelve hours night duty. The pupils have a right to one-half of Sunday, and are often given a half-day in the week. They are not placed on night duty for more than one month at a time, nor until three months after entrance. Vacations are given only during the summer, at Christmas and Easter holidays, as the work of the hospitals may permit, six weeks being the limit that may be given in three years' time. Time lost through illness, or absence on vacation over the six weeks' limit must be made up. In illness, pupils will be cared for gratuitously. The school is non-sectarian, and there are no religious services connected with it except morning prayers. Upon the honourable and successful completion of her course the graduate receives a diploma signed by the examining board and by a committee of the board of managers.

THE THREE YEARS' TRAINING.

The schedule of time for three years is seven months medical, five months surgical, three months operating rooms, six months private wards Presbyterian Hospital, two months children, two months obstetrical, six weeks laparotomy ward, three months special nursing of private patients, five months as head nurse, and six weeks vacation.

During the third year the theoretical work done is entirely preparation for work after graduation, the first being public hygiene, ventilation, heating, lighting, drainage, garbage, water, ice, meat and milk supply, quarantine, &c., the method followed being that of medical societies and other professional clubs, each pupil preparing a formal paper open for discussion by the class.

Following comes a course of instruction by the superintendent upon hospital and training school administration and nursing ethics.

The alumnæ numbers about 600, the larger number doing private nursing in the various large cities, 60 occupying hospital and training school positions, and several being in the army at Manila. The school maintains a directory for its graduates who are nursing in Chicago, the registration fee being five dollars per annum, the nurse stipulating her own terms, usually from 20 to 25 dollars per week, and also stating the kind of work she prefers. There is no further expense to the nurse, and no fee is exacted from the patient.

CRERAR NURSING.

In connection with the graduates' directory a branch of work is carried on which is unique, known as Crerar Nursing, or providing graduated nurses to people of moderate means who are unable to pay full rates. Seven years ago the school came into a legacy of fifty thousand dollars bequeathed by Mr. John Crerar, and the board of managers, feeling that the school was indebted so largely to the public, resolved to use the income from this money for such a purpose. It is designed to help salaried and better-class working people who are in nowise dependents, but are unable to employ trained nurses at the usual rates, the charity patients being cared for by the public hospitals and visiting nurse associations. The patient pays into the Crerar Fund from seven to ten dollars per week as he can afford, and the nurse is paid 20 dollars per week. The amount paid by patients, added to the regular income, enabled six nurses to be constantly employed last year, the cases being mostly obstetric or the acute medical.

The work is eminently satisfactory to all concerned, and seems to be the best solution of a problem which faces all hospitals and training schools for nurses.

THE ALUMNÆ ASSOCIATION.

The Alumnæ Association of the school forms a very important part of every graduate's professional life. It is second in size in this country, and has been very active from the beginning. Systematic work and study is constantly done, not only in the immediate affairs of the society, but in all public questions which in any way concern nurses or nursing. A room for sick nurses has been endowed in perpetuity at the Presbyterian Hospital, beside the maintenance of a sick benefit fund for those who are out of the city or unable to enter the hospital. The society meets the first Thursday in every month but July and August, the time being devoted to formal papers and discussions, the report of each meeting being printed and sent to every member. These reports also contains news items about, and letters from, members from all quarters of the globe, and are found to be a most satisfactory and enjoyable means of keeping up school relations. Afternoon teas and social evenings occur semi-occasionally, but on the day of graduation—the first Tuesday in June, a formal banquet is given to the graduating class, which brings members from far and near, and shows a splendid body of intelligent women, public-spirited, enthusiastic, and bound together by a kindred tie.

The policy of the school has always been rather conservative, the rapid growth and development being thrust upon it rather than sought; the desire being throughout to foster a healthy, steady improvement in providing, as far as possible, for the thorough practical education of the young women entrusted to its care, feeling that by so doing not only will the sick benefit by it, but the community must be the better for every educated, intelligent nurse occupying an honourable self-supporting position.

To those who share the belief of William Morris in his doctrine of work for hands as well as head and heart, the modern trained nurse must ever be a source of gratification.

### The Imperial Yeomanry Nurses at Devonshire House.

On Friday last the Prince of Wales inspected the "personnel" of the Imperial Yeomanry Hospital on the eve of its departure for South Africa. The 80 orderlies of the St. John Ambulance Society, in charge of Colonel Sloggett, were paraded first before his Royal Highness, who addressed them with encouraging words. They then gave place to the nursing staff who, headed by their superintendent, Miss Fisher, and night superintendent, Miss Preston, and numbering about 40, were individually presented by name by Mr. Fripp to the Prince. After shaking hands with the two superintendents his Royal Highness genially acknowledged the curtsies of the nurses. The latter passed on to the beautiful suite of rooms leading out of the ballroom, where, through the kindness of the Duchess of Devonshire, they were enabled to enjoy the wonderful art treasures on the walls. The medical staff were next presented to his Royal Highness, while tea and coffee were served to the nursing staff in another room. After being photographed, the nursing staff departed, having enjoyed their reception exceedingly. As already announced, they leave for South Africa in the "Guelph" on Saturday.

Photo by the Photographic Bureau, Upper Norwood.

### Death in Our Ranks.

We regret to announce the death of Miss Kate Treadaway from pneumonia, on February 3rd. She had been probationer at the Poplar and Stepney Sick Asylum for over twelve months, and, we are informed, had endeared herself to all her colleagues. A memorial service was held in the hospital chapel on Monday, February 5th, the funeral taking place on Thursday, the 8th, at Tooting Cemetery. Several of the staff from the Fountain Hospital, where Nurse Treadaway had been for three years, were present, as well as some from the Poplar and Stepney Sick Asylum.

### Appointments.

Lowestoft Convalescent Home.—Miss Stephanie Harvey, the new Matron, writes to state that she was matron at the Easingwold Hospital, Yorkshire, and assistant matron at the Metropolitan Convalescent Home, Walton-on-Thames, for one year.

### Where to Go.

London Hospital.—Conversazione, eight to eleven p.m., Thursday, February 22nd.

### Echoes from the Outside World.

AN OPEN LETTER TO A HOSPITAL NURSE.

The fact that Lord Roberts is now in command of the British forces on the Modder River is more significant than the third retirement of General Buller across the Tugela. One does not, of course, like to hear of these retirements, and they mean the further postponement of the relief of Ladysmith, which we all desire so keenly; but in this case the withdrawal was evidently wise policy, and saved a useless sacrifice of human life. Lord Roberts, we may be sure, would not himself have taken over the command at Modder River unless a great effort in that direction had been contemplated. But, though our interest in the operations there has thus been quickened, we may evidently have to wait some time longerf or news of definite results. Meanwhile, there are two personal incidents worth noting. One is the discovery, by Lord Kitchener, of the debt which Mafeking and England owe to the gallant son of the Prime Minister. It appears that when, on the outbreak of the war, Lord Edward Cecil heard the extent of the order given to the Government contractor in respect to stores for Mafeking, he at once, on his own responsibility, directed that the quantity should be increased fourfold, pledging his private credit for the amount. The guarantee was readily accepted, and it is entirely owing to the forethought of Lord Edward that Mafeking has so far enjoyed an abundance of provisions, and can, it is stated, hold out until June. The manner in which the Boers conduct the campaign often calls for criticism, but don't you think it was kind of General Joubert to allow a wife to pass through the enemy's lines in order to reach her suffering husband at Ladysmith? If Major Doveton recovers he will always be grateful for the consideration shown; and if not, the Boer General will have the satisfaction of knowing that he was the means of soothing the last moments of an honourable opponent in the way the latter most desired.

Women, I suppose, are not expected to understand the details of the military scheme which was laid before both Houses of Parliament this week, but we do at least grasp the essence of it, which is that, while the Army is to be strengthened, there is not to be any introduction of the principle of compulsion. Our young men are not to be forced to act as defenders of their country; they are to be encouraged, by the offer of various advantages, to don the Queen's uniform. These advantages, especially in the case of the volunteers, are to be of a solid character, though one does not quite see how some volunteers can manage to go under canvas for a period of training. Of course, the military experts are grumbling about the shortcomings of the scheme, and I dare say that there are shortcomings—in such a case they are inevitable—but the general opinion is that the Government are doing as much as could be expected in present conditions.

It must surely be wrong that money which is subscribed by the public for charitable purposes should be hoarded up by the persons who are supposed to administer it. What would be said if, after the Lord Mayor of London had raised a Mansion House Fund of large dimensions, for national purposes, as on the present occasion, only a portion of it reached the individuals for whose benefit it was freely contributed? To some extent this has really, it seems, been done in the past, and over £200,000 of the existing War Fund has been handed over to the Royal Patriotic Commission. But the cardinal principle of the Commission appears to have been to accumulate money which was placed in their charge to distribute. No one accuses them of wrong-doing in any shape or form. They are able to give an account of their stewardship. But they have made the mistake of concealing the coin which was meant to alleviate human misery and distress. In this case, big balances and profitable investments are not wanted, but rather the avoidance of balances and the exhaustion of capital. The condemnation of the past policy of the Commissioners lies in such facts as this—that they possess a balance of more than £600,000 of the Crimean Fund in their hands, while our brave soldiers who fought in that campaign have in some instances died in the direst state of poverty. If the pockets of the generous are not to be closed to future appeals, such a condition of things must be altered, and nurses will share the hope of those who, relying upon the promise of Mr. Balfour, look for speedy and decisive action on the part of a committee of inquiry appointed by the Government.

Sweet-tempered as the Duchess of Connaught is known to be, she must have been somewhat annoyed to notice that rumour has been busily engaged in assigning the youthful hand of Princess Margaret to the German Crown Prince. Apart altogether from this consideration, which would seem to render the engagement suggested highly improbable, it is not at all likely that, at present, the Princess has been concerned about the question of a matrimonial alliance. It does not by any means follow that because one Royal lady has always favoured the idea of very early marriages for her daughters, that her sisters-in-law share her views. The probability is that the Duke and Duchess of Connaught hope and expect, in spite of her attractions, to keep their daughter at home for some time to come.

As Sir Edward Clarke does not approve the policy of the Government in respect to the war, and the Plymouth people are strongly in favour of it, he has made way for a man who will give effect to their views; and the Hon. Ivor Guest is to succeed him without a contest. The curious thing is that, as Mr. Guest is off to the front, Plymouth will, as a matter of fact, still be minus a second member as long as the war lasts. But what one admires about Sir Edward Clarke is that he has not permitted the incident to convert him into a bitter adversary of the party with which he has always been identified. He took the chair at the annual meeting of the Holborn Conservative Association on Monday night as if nothing had happened, was re-elected president almost without a dissenting voice being raised, and has not said an unkind word about any of his old associates. There must be room in the House of Commons for a politician of this stamp, and when the war is over and the foundations of permanent peace are securely laid, I imagine that Sir Edward Clarke will be welcomed back by both sides. His action has been in marked contrast with two or three members who stick to their seats in spite of the protests of their constituents.

Have you observed that a new sphere of work has been opened for the up-to-date woman? She must, however, be very up to date, if she is to fulfil the conditions laid down by a gentleman "preparing for Parliament." This personage requires, though not before May, "at his country place," the services of "a cultured, well-bred lady, competent to assist him in political work and social duties." He offers, it must be confessed, fairly good remuneration, though if the lady is expected to coach him for the duties of a member of Parliament, "about £400 a year" is not excessive. The highest references are not only required, but will, it is stated, also be given. Hitherto the position of private secretary to a member of Parliament, or at any rate to a Minister of the Crown, has been practically sacred to the male sex, but this looks as if we might be on the eve of poaching on yet another of the preserves of the unfortunate males.

### A Book and its Story.

THE TOPIC OF THE HOUR.

At this moment, when the thoughts, the hearts, and the prayers of the British nation are centred on the country to which daily all that is best and bravest of the manhood of the empire goes forth to fight for, and to uphold, the supremacy of the British flag against an obstructive and oppressive foe, any literature relating to South Africa, apart from the subject of the war itself, is eagerly read. A reprint of "Side Lights on South Africa"* by Roy Devereux appears opportunely. It gives a clear and vivid description of the position of affairs, with numerous pen-portraits of the men who direct and control them in that, at present, unhappy country. Foremost amongst these is President Kruger. In an unpretentious bungalow dwelling, lacking in all the elements of state associated with one holding so important a position, "Oom Paul" receives without ceremony those desiring audience. The bare, sparsely-furnished room in which he presides is suggestive, in its stiff, inartistic interior, of "an English hostelry during the hideous mid-Victorian reign of horsehair and mahogany." From the end of this room rose the ponderous form of the President on the occasion of an interview which the author had with him. His eyes were obscured by dark blue spectacles, so it was impossible to see their expression. But they are eyes in which cunning and greed is the dominant expression, if we may judge from his portraits. The audience was brief, and Kruger's replies, through the medium of an interpreter, to a few simple questions, "vague and parabolic." There was a characteristic lack of dignity about the proceedings in the repeated appearance of other visitors "pushing open the door and retiring only on seeing a stranger within." The impression left on the mind of the visitor was one naturally of irritation "that the assumption of an absolute authority should be so shorn of all those attributes which grace a tyrant, if they do not justify him. The spectacle of an ignorant peasant imposing a vexatious rule over an educated multitude strikes one as a relic of barbarism. . . . His strength is to sit still. . . . Nothing like it can be found in the history of modern times. There was nothing to compare with it in mediæval days." In spite of his ignorance, lack of imagination, his rigid Calvinism, which does not prevent his accepting bribes from any alien who offers them—for "Did not the chosen of the Lord spoil the Egyptians before they destroyed Pharoah's hosts?"—in spite, also, of a total indifference to the movement and stress of modern life, "which sweeps past his adamantine composure without availing so much against it as the quiver of an eyelash, Kruger is regarded by all sections of his most ignorant countrymen, except the semi-educated official class, with a sort of superstitious awe. And the younger generation of Boers, who disagree with his policy, either fear to oppose it or are powerless to do so, while the executive tolerates his despotic will because of what he has done for the land, believing that the end of his activity is not very far off." By which it is clear that a "United South Africa," with Oom Paul controlling, is a dream not likely to be fulfilled.

Sympathisers with the Boers have a mistaken idea that they are a race of simple agriculturists, leading a happy, pastoral life in their farm homesteads, having made the lonely veldt to blossom as the rose by their industrious cultivation and thrifty care. But the author as an eye-witness tells a very different story. She found on arriving at a typical Boer farm some miles from Pretoria, standing alone on the solitary veldt, a bare, white-washed house, surrounded by dilapidated outbuildings; no sign of crops or of ground in a state of cultivation, "only a neglected patch where mealies had once grown, whose ripening ears hung on the leafless branches of a discarded tender tree." None of the cheerful sights associated with a farm life at home was visible. The cattle graze far away on the veldt. A typical Boer of the "Dopper" class, whose immense frame suggested unusual strength in the past, but who, in his old age, had become unwieldy through inertia, sat smoking inside. The house was indescribably neglected and dirty, but with no suggestion of poverty or suffering, and is compared to "a labourer's cottage on the West Coast of Ireland." The Boer farmer has never taken advantage of the enormous increase of population following the discovery of gold to cultivate farm produce, for which there would be a ready sale with "a market at his very door," and which from the extraordinary fertility of the soil would be a quick and certain source of gain; on the contrary, "owing to his chronic indolence and indifference, all the farm produce consumed on the Rand has to be imported."

A coach journey with its attendant discomforts is graphically described. Thirty hours in a vehicle constructed to carry nine passengers inside, each sitting in a position of absolute rigidity, when full, was on this occasion equally uncomfortable from the lack of a complement of passengers, for "the caravan struggled painfully forward, oscillating violently from side to side like a ship on a stormy sea. . . . Inside darkness prevailed, but this darkness contained nothing that was synonymous with peace. We groped vainly after our flying baggage as the mules, urged by the driver's resounding whip to a supreme effort, brought the coach back to equilibrium after sinking deeply into the sandy track." The little town of Mafeking, to which the writer was bound, "has the tranquil, tidy, and conventional appearance, with a progressive air also, of an English country town, leavened with the decorous energy of a commercial centre."

"Pietermaritzburg set in its frame of undulating woodland is wonderfully reminiscent of Devonshire," and Durban is Devonshire also, "her leafy luxuriance bathed in sunlight, and her sky and sea like rain-washed forget-me-nots. But the flowers are tropical. In palm-fringed gardens oleanders and camellias flame against the false violet of creeping burgeonvillias and mingle with the waxen cups and shining leaves of the magnolia trees."

No book bearing the title of the one under discussion would be complete without copious notes on Cecil Rhodes. Writing of his colony, Rhodesia, the author declares that, in comparison with New South Wales—whose expansion cost the ancestors of those who carp at the great powers wielded by the Chartered Company £10,000,000, and was stigmatised by its early governors as a worthless encumbrance, while to-day its trade is valued at £100,000,000, and its inhabitants number 4,000,000—Rhodesia has cost the British taxpayer nothing, and may in future bring as much as New South Wales to the Imperial Treasury. So much has been written of the "Great Man" or the "Arch Fiend," according to the point of view at home, that little remains that is new to be said. But in "Sidelights on South Africa" there are glimpses of him in his public and private capacity which bring vividly home to the reader the complex, versatile genius of this leader of men. No book that has been recently written brings more forcibly and tritely before us the political, social, and physical difficulties of the situation in South Africa. It is a book to be read, certainly.

* "Side Lights on South Africa." One vol. By Roy Devereux. (Sampson Low, Marston, and Co. London. 6s.

### Novelties for Nurses.

A NEW STOCKING SUSPENDER.

THE "Portia" Stocking Suspender and Shoulder Support is a new and ingenious contrivance. It is very simple in arrangement, and consists solely of stout elastic and the necessary buckles and clips. It will be found useful for ladies, and suitable also for the use of little boys. The illustration will afford the best explanation of the application of the contrivance, which can be secured by all drapers from the "Portia" Company, 202, Romany Road, West Norwood.

BEEHIVE WOOLS.

(MESSRS. J. AND J. BALDWIN, HALIFAX.)

THERE is a great sale in knitting and crochet wools just at present, when the demand for warm garments for our soldiers is so great, and khaki-coloured wools are especially popular. It is a great advantage to secure the best materials, and in this connection we have no hesitation in saying that J. and J. Baldwin's "Beehive" wools are of superfine quality, and should be asked for when purchases are about to be made. The range of choice is immense, and the exquisite softness and evenness of finish in some of the varieties cannot fail to provoke the admiration of the connoisseur. Khaki shades are produced in all the different qualities. It is difficult to select any one wool for special notice where the excellence is so uniform. All the Scotch fingerings are most tempting, whilst amongst the other varieties we would call attention to the even, firm, and soft quality of the soft knitting wools, and the remarkable fineness of the Pyranees wool, which might be utilised to fashion a veil. The silk vest wool is very effective. Many of the wools are supplied in four different plies.

### St. George's Infirmary.

MEETING OF GUARDIANS.

THE Guardians of St. George's Union, at their board meeting on Wednesday, had before them the report of the Visiting Committee of the St. George's Infirmary, in which it was stated that "the committee had had under consideration certain statements which had been made in THE HOSPITAL and elsewhere; some of these had been found to be entirely without foundation, and others had already been dealt with. The rest, which were minor matters of detail, the committee were dealing with in the ordinary way." Upon this report considerable discussion was raised, and the various charges made were gone into in some detail. It was denied that the patients' food was either bad in quality or short in supply, the scale being that laid down by the Local Government Board, plus extras as ordered by the doctor; while it was claimed that the intervals between meals were not so great as at some of our best hospitals. Similar statements were made regarding the nurses' food. As to the question of damp clothing, it had happened that once the dresses had been delivered from the laundry unaired, but their laundress superintendent would not receive them, and they were sent back. It was admitted that there were no separate wards for children; that at night there was but one nurse to two wards; and that, except the staircase, there was no means of communication between the wards. In respect of these things, however, they hoped to be much better off when the new buildings, which were to cost something like £100,000, were completed. Most of the speakers expressed either resentment or regret that the matter should have been made public at all; indeed, the attitude of the Guardians generally was summed up in the letter addressed by the clerk to the lady visitor, through whom attention has been drawn to what is complained of. This letter, after stating that full consideration had been given by the committee to the points to which their attention had been drawn, that several statements had been found to be inaccurate; that others referred to matters which had already been dealt with or were still in process of alteration, concluded with the expression of opinion that the committee "did not consider it necessary to discuss the questions with a lady visitor whose privileged place in the infirmary was at the bedside of patients."

### Minor Appointments.

WARRINGTON INFIRMARY.—Miss F. M. Robinson has been appointed Staff Nurse. She was trained at Southport Infirmary for three years.

FULHAM UNION.—Miss Emma Bell has been appointed Assistant Nurse. She was trained by the Meath Workhouse Attendants' Association at the Crumpsall Infirmary, Manchester.

LOWESTOFT HOSPITAL.—Miss Agnes Moffat has been appointed Charge Nurse. She was trained at Worcester General Hospital for three years, and has done some private nursing.

ROTHERHAM HOSPITAL.—Miss Mary Howell has been appointed Charge Nurse. She was trained at Swansea General Infirmary, and has since been sister at the Stroud Hospital for two years.

DORSET COUNTY HOSPITAL, DORCHESTER.—Miss Myra Simons has been appointed Charge Nurse. She was trained at the Bristol General Hospital, and has since been engaged in private nursing for two years.

HARTLEPOOLS HOSPITAL.—Miss R. Moody has been appointed Sister of male landing and operation theatre. She was trained at the Huddersfield Infirmary, and has since been charge nurse at the Dover Hospital and sister of the Monsall Fever Hospital.

SHEFFIELD UNION WORKHOUSE HOSPITAL.—Miss Maria Jane Scanes has been appointed Charge Nurse. She was trained at Chorlton Hospital, Manchester; and has been charge nurse at Kingston Infirmary; night nurse lying-in ward St. Pancras Workhouse; and charge nurse at Battle Infirmary.

ALLT-YR-YN HOSPITAL, NEWPORT, MON.—Miss Harriet Williams has been appointed Sister. She was trained at the Kennedy Street Hospital, Glasgow, and for the last three years has been staff nurse at the Allt-yr-yn Hospital. Miss Gertrude Hastings has been appointed Sister. She was trained at the Kensington Infirmary and City Hospital, Edinburgh, afterwards filling the posts of sister in charge of typhoid wards at the Sanatorium, Hull, and nurse-matron the Hospital, Hinckley.

### Everybody's Opinion.

[Correspondence on all subjects is invited, but we cannot in any way be responsible for the opinions expressed by our correspondents. No communication can be entertained if the name and address of the correspondent is not given, as a guarantee of good faith but not necessarily for publication, or unless one side of the paper only is written on.]

### THE AGE LIMIT.

"Lady Superintendent" writes: I must express my appreciation of the excellent paper by Miss Mary Gardner on the age limit and the commercial element in nursing. I am quite convinced that if this point could be driven home more we might get the evil remedied. It is really cruel to put the age limit at forty. I think that it should be extended ten years. Certainly women have in many instances been much to blame. They should exert more self-control over themselves, and not give way to emotional feeling and craving for sympathy during a period which is perfectly natural. It is these selfish ones of our sex who have laid a burden upon others.

### DANCES IN HOSPITALS.

"A Matron" writes: "A Loyal Nurse" will find as she goes on that she has more and more to learn. The most capable and best-trained nurses are often the most diffident as to their powers, because they have a fuller grasp of the whole position. Hospitals are not governed in the best way by seeking popularity in pleasing the nursing staff, but in holding up a high ideal of training and in not allowing an institution meant for the sick and dying to be wrongly used. And when hospitals are situated in towns, where there are facilities for nurses to enjoy concerts, theatres, &c., and occasional leave of absence for a dance, given by their own friends, there is no justification for holding one inside a hospital. Would our great leader and pioneer in nursing—Miss Florence Nightingale—advocate such a thing? Let us try and get back to more of her spirit in our profession. "We needs must love the highest when we see it."

### CHANGES AT A NORTH LONDON INFIRMARY.

"S. G." writes: Can you spare me a small space in your paper, which has done so much for the nurses? An infirmary in the North of London, containing between four and five hundred patients, appears to be in need of reform. Surely there must be something greatly wrong when about twelve nurses leave in three months, some of whom were probationers who only stayed their trial month. Everyone lives in a state of uncertainty, and there is always grumbling, and not without occasion. On night duty there are three nurses to over two hundred patients (seventy-two on a flat), and as there are many bad cases, it is quite impossible for one nurse to give as much attention as is needful. On day duty one is in a particular ward to-day and another to-morrow. This cannot be good for either the sisters, nurses or patients. It is impossible to take an interest in your work when you are constantly being moved. Then, as regards meals, one might as well be deaf and dumb. If, on entering the mess-room, you should laugh and talk a little, you are asked to "make less noise." One never knows the rules of the place until (through ignorance) one gets a reprimand for breaking them. Talk about "red-tapeism"—Government is not in it. We must have written "orders" for every little thing. Passing to a smaller matter, do you consider it quite the thing for servants and nurses to dress alike? It is bad enough when all wear the same caps, but one draws the line at dresses.

### THE COMMERCIAL ELEMENT IN NURSING.

"An Ex-Paying Pro." writes: In a recent number the tendency to make the commercial element too prominent in nursing was deplored by "A London Matron." All nurses will agree that to make the getting of money the chief object is a very low ideal for a nurse. But as the remuneration for nursing is the worst of any profession, surely this could scarcely be an inducement for nurses to take up the work. I do not for a moment say but that nursing is taken up for the most varied motives, *i.e.*, disappointment in love, a desire to get rid of home ties, a desire for a new experience or for scientific work, a wish to get on, and the necessity to do something to earn a livelihood. Last and best is the wish to serve God, by helping those who need care. I have been in four hospitals—two in England and two in Scotland. In the two former the nurses paid premiums for the first year; in one Scotch they paid as probationer for a term of months. In the other no probationer paid. The moral tone in the Scotch hospitals was quite as good as in the English. In the last I mention money neither helped nor hindered a nurse from coming. She was taken because she was considered a fit woman by the matron. She was on trial for three months, after which she was either asked to stay or not, as was thought best for the institution. We must remember that hospitals were made for the sick, and not for nurses. The sick are the first to be thought of and their comfort. And let us take the best women we can get from all parts and all quarters and all classes. Why, because a woman has £200 or £500 a year, should she be debarred from following work she loves? No other profession is so narrow as to say we will only gather from one source. Take all and give them a chance. Do not keep the rich one any more than the poor one, if she does not do well. Hospital life banishes all feeling of distinction of class. It is like an army shoulder to shoulder, fighting against sorrow, suffering, and sin. One nurse will do the brain work best; she will help another, who may, in her turn, show her better how to do the manual work. Then the woman of leisure may have seen places and things of interest, and may be able to refresh the tired nurses by a talk of foreign lands, and stop the conversation of "shop"; or she may have a trained voice which will delight nurses and patients as she sings the evening hymn. May I add that my ideal of a perfect nurse is a perfect woman—gentle, loving, obedient, patient, with self-control and self-respect.

### ST. GEORGE'S INFIRMARY, FULHAM ROAD.

"Two of the Nurses" write: We regret the tone you have adopted in speaking of the matron at this infirmary, the report of whose resignation, by-the-bye, is somewhat premature. Leaving other nurses to speak for themselves, we are proud to say that we are being trained under her, her presence here being the chief inducement to put up with the long hours, straitened accommodation (in which she also shares), and overwork, of which we have complained. We are quite sure that you do not know our matron, or you would not speak so slightingly of her. But why bring the matron's name into the question at all, when by your own showing it is the guardians who are responsible for the present state of things? With regard to her own part towards the patients, nothing can be more marked than her consideration and thoughtfulness for them. The wards are visited frequently by her, and minute inquiries made with regard to each patient, particularly in relation to meals. A frequent question is, "Does he take his food?" "If not, is it saved for him in case he would like it later on?" Here we would say that a nurse is privileged in serious cases to portion out the patient's allowance as she sees fit. In a bad pneumonia, for instance, she would divide the allowance of beef-tea and milk into feeds, distributing them evenly for the twenty-four hours, so that the strength of the patient should be kept up by constant nourishment. The careful feeding of the patients is one of the points on which the matron strongly insists, and she is quick to note any signs of neglect in this or any other duty. One of us at least can testify that the matron has frequently given up her half-days and sleeping time for the welfare of the patients, putting up sweets and toys for the children at Christmas-time till one o'clock in the morning, and in the absence of the night superintendent through illness, instead of delegating her duties to others, staying up night after night till two or three o'clock, so that the nurses should feel that they could send for her, if in need of advice or assistance; and visiting every ward where there were bad cases, last thing before retiring, to inquire after them. The need of more nurses is a point which the matron has strongly represented to the Board of Guardians ever since she has been here. On this point, at least, instead of lamenting that nothing has been done for the "poor patients," we can rejoice that more nurses mean that the "shaking of feeble old women" or the "striking of little children" will then be an impossibility.

### For Reading to the Sick.

The Lord shall give thee rest from thy sorrow and from thy fear, and from the hard bondage wherein thou wast made to serve.—*Isa.* xiv. 3.

Gethsemane
Denied our Lord all human sympathy!
And deepest grief
Is what we bear and love for others' sake,
Smiling the while lest other loving hearts should break
For our relief!
O hearts that faint
Beneath your burdens great, but make no plaint,
Lift up your eyes,
Somewhere beyond, the life you give is found,
Somewhere, we know, by God's Own hand is crowned
Love's sacrifice. —*M. Drake.*

**Reading.**

Happy is the soul which offers itself to its Lord, every time it is troubled and disturbed. And if the struggle last long, and you cannot as quickly as you would wish bring your will into conformity with the will of God, be not on this account discouraged or bewildered: persevere in self-oblation and prayer, and you shall gain the victory.

Look at Christ's conflict in the garden, and how his humanity recoiled from it, saying, "Father, if it be possible, let this cup pass from me." But at once He placed His soul in solitude, and with a will free and detached, said with deep humility. "Nevertheless, not My will, but Thine be done." "See and act according to this pattern." Do not move a step, when you find yourself in any difficulty, until you have raised your eyes to Christ on the Cross; for there you will see, written and stamped in large characters, how you ought to act. Copy faithfully this example. Be not dismayed, if sometimes your love of self disturbs you; do not leave the cross, but return to prayer, and persevere in lowliness till you have lost your own will, and will only that God's Will may be done in you. And if, when you leave off praying, you have gathered only this one fruit, be contented; but if you have not achieved this, your soul will remain empty and hungry. Strive not to brood over any thing, even for a short time, but to let God alone dwell in your heart.—*F. de Sales.*

Once again to wake nor wish to sleep,
Once again to feel, nor feel a pain!
Rouse thy soul to watch and pray and weep,
Once again.
Hope afresh, for hope shall not be in vain,
Start afresh along the exceeding steep
Road to glory, long and rough and plain.
Sow and reap; for while these moment creep,
Time and earth and life are on the wane;
Now in tears; to-morrow laugh and reap
Once again. —*C. Rossetti.*

### Presentation.

City of Dublin Nursing Institution.—The nurses of the City of Dublin Nursing Institution are about to present their lady superintendent, Mrs. Jane Kildare Treacy, with a fine half-length portrait in oils, the size of life, of the distinguished Irish surgeon and vice-chairman of their board of directors, the late Mr. William Ireland de Courcy Wheeler, F.R.C.S. The picture, which has been executed by Mr. Catterson Smith, R.H.A., the eminent Irish portrait painter, is a striking likeness as well as a choice work of art, and is the second token of esteem and regard which Mrs. Treacy has received from her working staff. It is to be exhibited at the Royal Hibernian Academy's annual exhibition, after which it will be hung up in Mrs. Treacy's business room at the institution.

### Notes and Queries.

**The contents of the Editor's Letter-box have now reached such unwieldy proportions that it has become necessary to establish a hard and fast rule regarding Answers to Correspondents. In future, all questions requiring replies will continue to be answered in this column without any fee. If an answer is required by letter, a fee of half-a-crown must be enclosed with the note containing the enquiry. We are always pleased to help our numerous correspondents to the fullest extent, and we can trust them to sympathise in the overwhelming amount of writing which makes the new rules a necessity.**

**Every communication must be accompanied by the writer's name and address, otherwise it will receive no attention.**

*"The Absent-minded Giver."*

(198) In reply to several inquiries, "The Absent-minded Giver" was originally published, as mentioned at the time, in *To-Day*, and all applications about it should be made to the Editor of that paper, 89, Essex Street, Strand, W.C.

*Young Probationer.*

(199) I should feel very much obliged if you could tell me any hospital that I could enter as probationer. I am 18 years of age, and desire to become a nurse.—*F. M.*

Apply to the matrons of such institutions, or see "The Nursing Profession: How and Where to Train."

*Monthly Nursing.*

(200) I want to learn monthly nursing, and to obtain a certificate from a good London school. I should like to know what is the cheapest way to obtain it, as I have to study economy. Can a person of ordinary education grasp the subject and obtain a certificate, or is it too difficult? What age is the best? In taking temperatures, why do some say that the mercury should be shaken down below 98·4 deg., and others below 95 deg.?—*Clara.*

A three months' training at a special hospital is usually enough to enable one to qualify for the certificate of the London Obstetrical Society. A thermometer should be shaken down considerably below 98·4, say to 95.

*Handbook on Nursing.*

(201) Would you kindly tell me whose medical and surgical handbook is considered the best? Have got Honnor Morten's, but wish for a more advanced and the latest edition.—*Reader.*

See list at foot of this column. Dr. Watson's handbook is the latest.

*Dispensing.*

(202) 1. How can a trained nurse qualify as a dispenser? 2. What would be the cost of necessary training? 3. Would she be likely to get employment? 4. What salary is usual?—*A. J., A. E. G.*, and *G. P.*

1. Apply the Secretary, the Pharmaceutical Society, 17, Bloomsbury Square, W.C. 2. Fee for apprenticeship £50 to £70. 3. If highly qualified, yes. 4. The salaries for dispensers are from £100 to £150, for assistants £40 to £60.

*Faulty Water Beds.*

(203) Will the editor kindly tell me if old water beds which leak are of any value as old rubber, and if so who will purchase them?—*Nurse Ellen.*

Write to the maker for information. Leaks can be repaired if not too bad.

*Home for Mental Nurses.*

(204) Will you kindly tell me if there is in London an institution for mental nurses where the nurses live in when not on a case?—*Alice.*

There is none that we know of, but the Nurses' Hostel, Francis Street W.C., and similar residential homes for nurses accept all *bona fide* nurses as boarders.

*Training School.*

(205) 1. What constitutes a training school as recognised by the Local Government Board for the post of superintendent nurse? 2. Would a certificate of proficiency in three nursing qualifications, signed by the medical officer and the matron (who is not a trained nurse), be accepted by the Local Government Board for the post of superintendent nurse?—*An Enquirer.*

The Local Government Board require for the post of superintendent nurse a nurse holding a three years' certificate from a training school for nurses maintaining a resident medical officer, but has declined to issue a list of recognised training schools. 2. Your qualifications are not in accordance with the Local Government Board Order.

*Health Visitor.*

(206) Will you kindly inform me what steps a trained and certificated nurse of ten years' experience should take to qualify as a health visitor?—*Amy.*

Apply the National Health Society, 53, Berners Street, W.

*South Africa.*

(207) Would you kindly tell me where a nurse should apply if desiring to go to the Transvaal?—*M. R. D.*

Apply to be placed on the Army Nursing Reserve, 18, Victoria Street, Westminster.

*Standard Books of Reference.*

"The Nursing Profession: How and Where to Train." 2s. net.

"The Nurses' Dictionary of Medical Terms." 2s. 6d. net.

"Burdett's Series of Nursing Text-Books." 1s. each.

"A Handbook for Nurses." (Illustrated.) 5s.

"Nursing: Its Theory and Practice." New Edition. 3s. 6d.

"Helps in Sickness and to Health." Fifteenth Thousand. 5s.

All these are published by The Scientific Press, Ltd., and may be obtained through any bookseller or direct from the publishers, 28 & 29, Southampton Street, London, W.C.

## Figures and Tables

**Figure f1:**
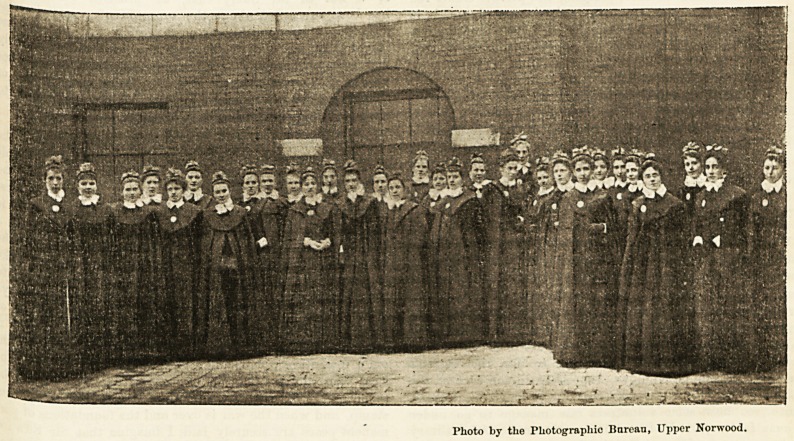


**Figure f2:**